# Unfertilized ovary pushes wheat flower open for cross-pollination

**DOI:** 10.1093/jxb/erx410

**Published:** 2017-11-30

**Authors:** Takashi Okada, J E A Ridma M Jayasinghe, Moureen Nansamba, Mathieu Baes, Patricia Warner, Allan Kouidri, David Correia, Vy Nguyen, Ryan Whitford, Ute Baumann

**Affiliations:** School of Agriculture, Food and Wine, University of Adelaide, Plant Genomics Centre, Hartley Grove, Urrbrae, SA, Australia

**Keywords:** Fertilization, flower, ovary, pericarp, pollination, wheat

## Abstract

Bread wheat is strongly autogamous; however, an opportunity for outcrossing occurs when self-pollination fails and florets open. The first phase of floret opening at anthesis is short and induced by lodicule turgidity. Some wheat florets re-open post-anthesis for several days, known as the ‘second opening’, for which the underlying mechanisms are largely unknown. We performed detailed physiological, anatomical, and histological investigations to understand the biological basis of the flower opening process. Wheat florets were observed open when the ovary was unfertilized. Unfertilized ovaries significantly increased in radial size post-anthesis, pushing the lemma and palea apart to open the florets. The absence of fertile pollen was not directly linked to this, but anther filament elongation coincided with initiation of ovary swelling. The pericarp of unfertilized ovaries did not undergo degeneration as normally seen in developing grains, instead pericarp cells remained intact and enlarged, leading to increased ovary radial size. This is a novel role for the ovary pericarp in wheat flower opening, and the knowledge is useful for facilitating cross-pollination in hybrid breeding. Ovary swelling may represent a survival mechanism in autogamous cereals such as wheat and barley, ensuring seed set in the absence of self-fertilization and increasing genetic diversity through cross-pollination.

## Teaser

Wheat flower opens when self-pollination fails. The unfertilized ovary pushes the palea and lemma apart by increasing radial size associated with cell enlargement and suppression of pericarp degradation, therefore facilitating cross-pollination.

## Introduction

Bread wheat (*Triticum aestivum* L.) is an important staple crop for human nutrition and it is the third highest crop after maize and rice in world production (FAO, 2015). Wheat is a strong self-pollinating (autogamous) plant, and outcrossing rates in wheat are typically low (<1%), with most seed produced by selfing (Griffin, 1987; Martin, 1990; Hucl, 1996). Autogamy is one reproductive strategy of plants to ensure seed production, which can be advantageous when pollinators and potential mates are scarce (Jain, 1976; Herlihy and Eckert, 2002). Cleistogamy, closed pollination associated with floral morphology and/or pollination behaviour, also helps self-pollination (Frankel and Galun, 1977). These traits are beneficial for agricultural cereal crops to ensure stable yield by minimizing environmental effects on pollination. Outcrossing cereal crops, such as corn and rye, are wind pollinated, and success by wind pollination is highly dependent on prevailing weather conditions. In contrast, only a small fraction of wheat seed is set by wind pollination. This relates to accumulation of cleistogamic floral morphologies and pollination behaviours in wheat, leading to self-pollination. Some of these traits are a likely consequence of wheat’s breeding and selection history. For example, introduction of the ‘green revolution’ semi-dwarf genes *Rht-B1*/*Rht-D1* into modern wheat cultivars not only prevented lodging and improved yield (Peng *et al.*, 1999), but also resulted in stiffer glumes with higher anther retention (Boeven *et al.*, 2016; Buerstmayr and Buerstmayr, 2016; He *et al.*, 2016). Wheat cultivars with no anther extrusion due to narrow flower opening or closed flowers also favour reduction or elimination of initial infection of Fusarium head blight (Gilsinger *et al.*, 2005; Kubo *et al.*, 2010). Each of these traits increases anther retention and consequently facilitates self-pollination and inbreeding.

Intensive inbred line breeding and developing modern wheat cultivars have led to narrowing genetic diversity within the germplasm pool due to the replacement of adapted landrace cultivars with modern inbred wheat cultivars (Charlesworth, 2003; Reif *et al.*, 2005). To overcome reduced genetic diversity and inbreeding depression, capturing heterosis is one of a few high priority strategies towards increasing yield and yield stability (Longin *et al.*, 2012; Whitford *et al.*, 2013; Okada and Whitford, 2017). Hybrid breeding requires converting predominantly autogamous wheat towards allogamy (outcrossing). Like many other plant species, wheat can adopt a mating system as a mixture of selfing and outcrossing (Barrett *et al.*, 1996; Barrett, 2002; Herlihy and Eckert, 2002), therefore providing an opportunity for outcrossing when self-pollination fails to occur. After the discovery of cytoplasmic male sterility in wheat (Kihara, 1951; Wilson and Ross, 1962), efforts towards establishing an efficient hybrid seed production system were initiated in the 1960s and have since had a long history (Curtis and Johnston, 1969; Driscoll, 1972; Pickett, 1993). However, hybrid wheat still remains a small fraction of current global production due to the high costs of seed production. This can be attributed to wheat’s strong self-pollinating nature. Therefore, it is important to understand flowering biology and pollination behaviour of wheat as it links directly to the yield and seed production for both inbred and hybrid breeding.

**Fig. 1.**
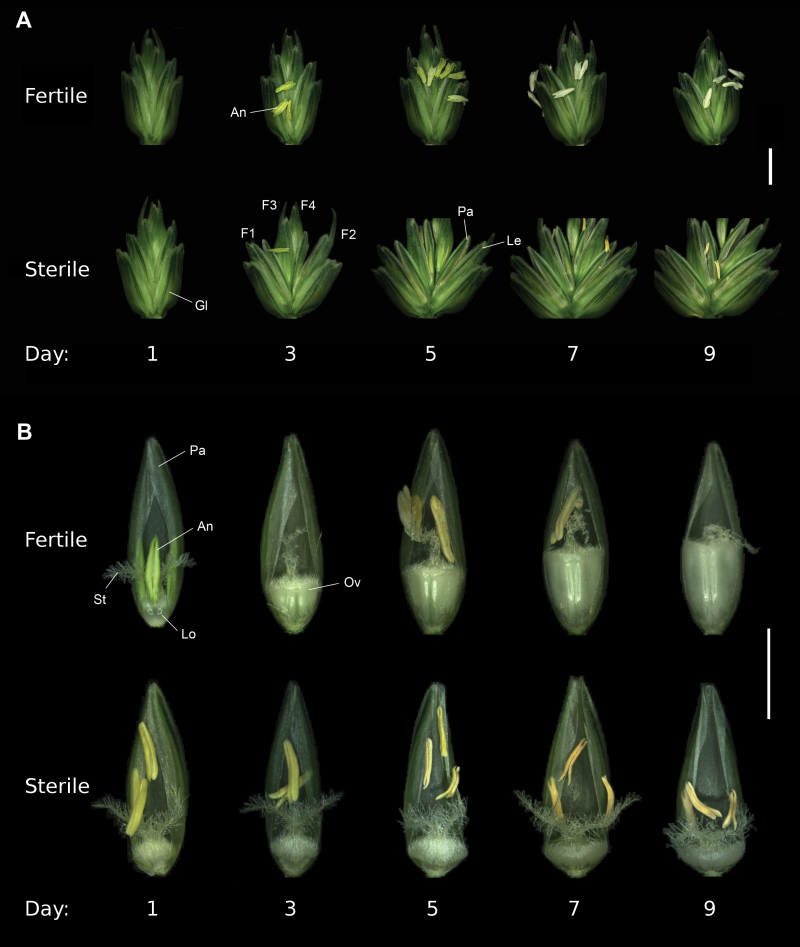
Spikelet and floret images of male-fertile and male-sterile plants during flowering time and early seed development. For sample staging, days were counted from full heading (spike completely emerged from the flag leaf), with the day of full heading being Day 1 (1 DF). (A) Spikelet of male-fertile (*Ms5*, top) and male-sterile (*ms5*, bottom) plants. (B) Floret of male-fertile (*Ms5*, top) and male-sterile (*ms5*, bottom) plants. Abbreviations: An, anther; F1–F4, floret number 1–4; Gl, glume; Le, lemma; Lo, lodicule; Pa, palea; Ov, ovary; St, stigma. Scale bars in (A) and (B) are 5 mm and 2 mm, respectively.

As in other cereal crops, the wheat inflorescence is referred to as an ear or spike, and consists of a main axis bearing a number of spikelets. Each spikelet has a short spikelet axis, two bottom glumes, and normally 2–5 fertile florets (de Vries, 1971). Wheat has hermaphrodite flowers. Each floret consists of a lemma and palea, and positioned between them are both male and female reproductive organs, including anthers, stigma, ovary, and lodicules, and all present at the base of the floret (Fig. 1; Supplementary Fig. S1 at *JXB* online). When lodicules become turgid, the lemma and palea are pushed apart, therefore opening the floret. Anthers begin to dehisce from the top while filaments elongate rapidly, and pollen falls within the floret and becomes lodged on the feathery stigma. Normally, anthers are pushed out of the floret, but the extent of anther extrusion is variable and dependent upon genotype and prevailing environmental conditions (de Vries, 1971; Frankel and Galun, 1977). Wheat accumulated with cleistogamic (closed) flower modifications such as stiff and large glume, stiff lemma, and small anthers during evolution and cultivation (Frankel and Galun, 1977; Whitford *et al.*, 2013; Nguyen *et al.*, 2015), resulting in a floral structure and pollination behaviour suited to selfing. Despite selection for inbreeding efficiency, variability in wheat’s outcrossing rate exists, with high outcrossing varieties tending to have a greater degree of spikelet opening (Hucl, 1996).

In barley and wheat, there are two stages of flower opening. The ‘first opening’ is at anthesis and associated with lodicule swelling as described above, and this is well known for many cereal crops (de Vries, 1971; Frankel and Galun, 1977; Virmani, 1994; Heslop-Harrison, 1996). This ‘first opening’ is rather short temporally, lasting only 7–36 min for barley (Kirby *et al.*, 1983) and 8–30 min for wheat (reviewed in de Vries, 1971; Pickett, 1993), and the duration of the ‘first opening’ is largely dependent on cultivars and weather conditions. After this time, florets close again as the lodicule collapses. Within a few days post-anthesis, some barley and wheat florets will open again, and this is known as the ‘second opening’ (Hoshikawa, 1960; Kirby *et al.*, 1983; Pickett, 1993). This time, the ovary not the lodicule is involved in the opening process. Enlargement of the ovary is reported to be the cause of the ‘second opening’, and in most cases the ovaries were unpollinated. However, there is limited knowledge about this process from early studies, and physiological and biological mechanisms associated with the ‘second opening’ are largely unknown. Furthermore, there is a lack of understanding of the flowering mechanisms for autogamous cereal wheat that lead to cross-pollination.

In this study, we aimed to investigate the anatomical and physiological mechanisms of the ‘second opening’ in wheat flowering and the biological link with other flowering processes. Observations in the flowering process were undertaken by measuring flower openness and ovary size in the male-sterile lines as well as emasculated male-fertile lines. Unfertilized ovaries significantly increased in radial size but showed only a slight increase in the vertical direction. This size increase exceeds the breadth of the lemma and forces the palea and lemma apart, resulting in an open floret. Suppression of pericarp degradation within unfertilized ovaries leads to the pericarp cells remaining intact and enlarging. We also demonstrated that barley ovaries swell when emasculated and remain unpollinated, but in a slightly different manner from those of wheat. Finally, we discuss the importance of cross-pollination in autogamous wheat.

## Materials and methods

### Plant materials

The bread wheat (*Triticum aestivum*) spring type variety Excalibur and male-sterile mutant lines *ms1* and *ms5* in the cultivar Chris background (Driscoll, 1975; Sasakuma *et al.*, 1978) were used. Plants were grown in growth room facilities (Narrabri Refrigeration & Air Conditioning, Narrabri, Australia) at the University of Adelaide, with temperature and daylight setting at 22 °C/15 °C (12 h day/12 h night: light on from 08.00 h to 20.00 h) for ovary size measurements, the emasculation experiment, and the filament elongation experiment. Additional plants were also grown in the glasshouse facility, in temperatures that ranged from ~14 °C to 24 °C and the day length maintained at ~12 h with the use of supplemental artificial lighting. Barley (*Hordeum vulgare*) cultivar Bowman, which has cleistogamic flowering behaviour, was grown in the field at the University of Adelaide, Waite campus (latitude 34°96'69''N, 138°63'38''E) from August to December 2016 under appropriate growth conditions.

### Measurement of ovary and floret traits

Spikelet samples were collected at different flowering stages from the plants growing in the controlled growth room. Sampling commenced when the spike had completely emerged from the flag leaf (Zadoks scale 59) (Zadoks *et al.*, 1974). The date of full heading and days from full heading were recorded for each spike and used to define the flowering stage of samples; for example, the first day of full heading was defined as 1 DF and 5 d from full heading as 5 DF. Whole spikelet images were taken as soon as spikelets were detached from the spike, and these images were used for angle measurements. Glume angle was measured between the tips of both glumes and the connection point to the spikelet pedicel, as shown by the yellow line in Supplementary Fig. S1B. Similarly, the floret angle was measured between the tips of lemmas of primary and secondary florets and the connection point to the spikelet pedicel (white line in Supplementary Fig. S1B). Florets and ovaries were dissected, and the ovary was placed at suitable orientations with the aid of the adhesive synthetic rubber Blu-Tack (Bostik Australia). The stigma was removed, and frontal and top ovary images were taken for measurements (Supplementary Fig. S1C). All the images were taken by using a stereo dissecting microscope (Leica MZFL III) with a DFC300 digital camera (Leica). Ovary length and angle measurements were manually performed by using image software FIJI (https://fiji.sc/).

### Emasculation experiment

Wheat cultivar Excalibur was emasculated at 1 DF for six spikelets located in the middle of the spike. Anthers of primary and secondary florets were removed by forceps; the rest of the spikelets were removed from the spike and then bagged to prevent cross-pollination by neighbouring plants. Subsequently, ovary samples were collected at 7 DF and images were taken for size measurement. Wheat cultivar Chris was used to investigate the effect of emasculation timing. At 1 DF, four middle spikelets located on one side of the spike were emasculated. At 3 DF, four spikelets located on the opposite side of the same spike were emasculated early in the morning by 08.00 h before anthesis, and untreated spikelets were removed prior to bagging. At 5 DF, ovary samples were collected for size measurements. For barley samples, the middle spikelets were emasculated, and spikes were bagged to prevent pollination. Seven days after emasculation, ovary samples were collected for imaging.

### Filament elongation experiment

Wheat male-sterile mutant lines *ms5* and *ms1* were used for a filament elongation experiment. Spikes of male-fertile and male-sterile plants were labelled at 1 DF. On 3 DF at ~11.00 h, primary and secondary florets of eight spikelets in the middle position were opened and visually checked to determine whether the anther filaments were elongated or not. If any one of the three filaments in the floret appeared to be elongated, it was regarded as a positive, and the number of positive and negative florets was counted. More than 17 spikes for each line from multiple plant samples (total number of spikes=86) were used for this experiment and plants were grown under controlled environmental conditions as described above.

### Histological observation of the ovary and microscopy

Ovary samples collected at different flowering times were fixed overnight in FAA (50% ethanol, 5% acetic acid, 4% formaldehyde), and then stored in 70% ethanol at 4 °C until processing. Ovary samples were dehydrated through an ethanol series and the samples were subsequently embedded in paraffin as described elsewhere. The 10 μm thin sections (cross or longitudinal) of ovary were prepared using a Leica RM2265 rotary microtome at Adelaide Microscopy Facility. The sections were stained by 0.05% toluidine blue solution (Soukup, 2014) and mounted with DPX Mountant (Fluka Analytical, Switzerland). Images of ovary sections were taken by using a Nikon ECLIPSE NiE optical microscope. The number of ovary mesocarp cells was counted from the longitudinal (ventral to dorsal) section images. A line was drawn at the maximum ovary depth position in the image, and the number of cells under the line was counted. For ovary mesocarp cell size, the perimeters of 10 randomly selected mesocarp cells at the top front position in the longitudinal sections were measured (in total, 80–110 cells per sample).

### TUNEL assay

Longitudinal ovary sections of 5 DF samples were used for TUNEL (terminal deoxynucleotidyl transferase dUTP nick-end labelling) assay to detect the signature of programed cell death (PCD). The TUNEL assay was performed as described (Gold *et al.*, 1993; Oberhammer *et al.*, 1993), with minor modifications. Ovary sections were deparaffinized and rehydrated according to the standard protocol. The sections were briefly washed with phosphate-buffered saline (PBS), fixed with 4% paraformaldehyde solution in PBS, and then washed with PBS again. Nick-end labelling was carried out by incubating slides with 10 U of terminal transferase (TdT; New England Biolab), 2 nmol of dNTP, and 0.25 nmol of digoxigenin-labelled 11-dUTP (Roche) in 200 μl of 1×TdT reaction buffer (New England Biolab) including 0.25 mM CoCl_2_ and 50 μg ml^–1^ BSA for 1 h at 37 °C. Following washing, antibody reaction and color detection by NBT/BCIP (nitroblue tetrazolium/5-bromo-4-chloro-3-indolyl-phosphate) were carried out as described (Okada *et al.*, 2006). The NBT/BCIP reaction was terminated after 3.5 min and slides were immediately rinsed for mounting. For the positive control sample, slides were treated with DNase I (2 U in 200 μl of 1×TURBO DNase buffer, Ambion) for 20 min at 37 °C prior to nick-end labelling. For negative controls, digoxigenin-labelled 11-dUTP was omitted from the reaction.

### Characterization of barley *HvVPE4* orthologues in wheat

The barley *HvVPE4* sequence (Radchuk *et al.*, 2011; accession no. FR696366) was used in a BLAST search against wheat genome sequence assemblies IWGSC and TGAC (https://www.wheatgenome.org) and the MIPS (http://pgsb.helmholtz-muenchen.de/plant/wheat/index.jsp) high confidence gene predictions (IWGSC, 2014, 2015) to identify wheat orthologues. BLASTN results identified three homeologous gene sequences in 5AL (TGACv1_scaffold_378334_5AL), 5BL (TGACv1_scaffold_407938_5BL), and 5DL (TGACv1_scaffold_434496_5DL) scaffolds with a significant E-value 0. Therefore, these homeologous sequences were designated *TaVPE4-A*, *TaVPE4-B*, and *TaVPE4-D*. *HvVPE4* and its wheat orthologue sequences were aligned using Geneious 9.1.5 (Kearse *et al.*, 2012). The deduced amino acid sequences for *TaVPE4* sequences were obtained and aligned with the HvVPE4 protein sequence using BioEdit (http://www.mbio.ncsu.edu/bioedit/bioedit.html) as shown in Supplementary Fig. S5. The phylogenetic tree of VPE (vacuolar processing enzyme) protein has been generated by using the maximum likelihood method based on the JTT matrix-based model (Jones *et al.*, 1992). The tree with the highest log likelihood (–6743.70) is shown in Supplementary Fig. S6. The analysis involved 31 amino acid sequences that include TaVPE4s and other plant VPEs (Radchuk *et al.*, 2011). All positions containing gaps and missing data were eliminated. Evolutionary analyses were conducted in MEGA7 (Kumar *et al.*, 2016).

### Expression analysis of wheat *TaVPE4* genes by RT–PCR

Total RNA was extracted from various wheat tissues using the ISOLATE II RNA Plant Kit (BIOLINE) followed by treatment with RNase-free TURBO DNase I (Ambion) according to the manufacturer’s instructions. After purification and quality check, 600 ng of total RNAs were used in a reverse transcription reaction with SuperScript III reverse transcriptase (Invitrogen) using oligo(dT) primers for cDNA synthesis. RNAs were extracted from three biological replicate samples for each tissue and cDNA was synthesized separately. The quality and quantity of cDNAs was analyzed for all individual cDNA samples by PCR with intron-spanning primers directed against wheat glyceraldehyde phosphate dehydrogenase (GAPDH; Sprunck *et al.*, 2005). After quality and quantity check, cDNA samples for each tissue were pooled for normalization and used for the gene expression study. Homeologue-specific primers for wheat *TaVPE4* genes were designed (Supplementary Table S1) by choosing specific single nucleotide polymorphisms for the 3' end of primers. The specificity of primers was investigated by using DNAs from nullisomic–tetrasomic lines (Sears, 1966) for wheat group 5 chromosome. PCR conditions were optimized (95 °C for 2 min, then 32 cycles of 94 °C for 15s, 59 °C for 30 s, and 72 °C for 30s) and a PCR band was confirmed to be absent in corresponding nullisomic lines for each homeologue-specific primer. *TaVPE4* gene-specific primers and *TaGAPDH* primers were used with various cDNAs for gene expression analysis. Primers for *TaVPE4-A* and *TaGAPDH* flank the intron region; therefore, the fragment size of genomic DNA and cDNA was different, while *TaVPE4-B* and *TaVPE4-D* contain no intron in the PCR amplicon, and thus there is no size difference between genomic and cDNA samples. PCR fragments were sequenced and it was further validated that they contained single sequences and were 100% identical to the genomic sequence.

## Results

### Male-sterile wheat flower opens after anthesis

**Fig. 2.**
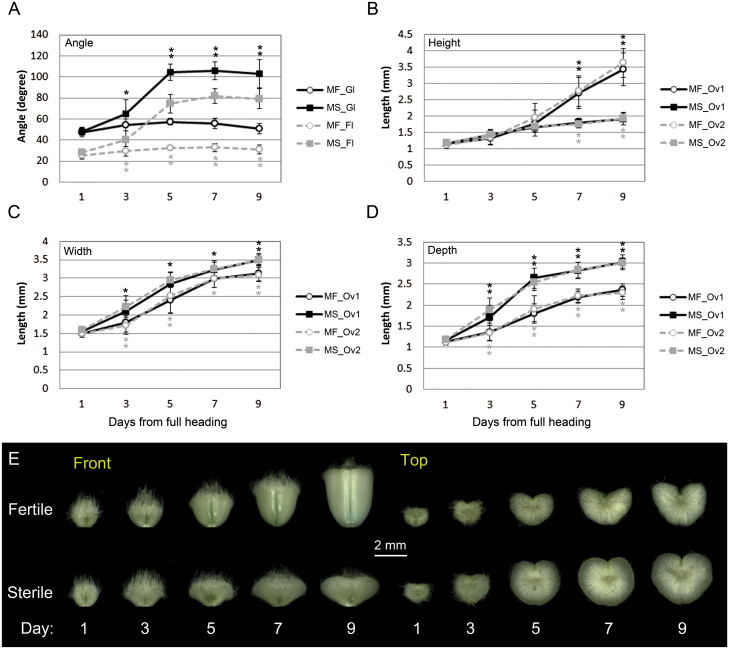
Measurements of flower opening angle and ovary size. (A) Glume angle (suffix, _Gl) and floret angle (suffix, _Fl), defined in Supplementary Fig. S1B, were measured at different flowering stages (1–9 d from full heading). Spikelets of male-fertile (prefix, MF) and male-sterile (prefix, MS) plants were used for the measurements. (B–D) Ovary height, width, and depth, defined in Supplementary Fig. S1C, were measured at different flowering stages. Ovaries from primary florets (suffix, _Ov1) and secondary florets (suffix, _Ov2) of male-fertile and male-sterile plants were used for the measurements. Significant differences between male-fertile and male-sterile plants for corresponding samples (indicated by black solid or grey dotted lines) at the same stage are indicated in the graphs (ANOVA test significant *P*-value: *P<0.01; **P<0.001). Error bars=SD. (E) Images of ovary/caryopsis during flowering and early grain development. Front and top images were taken as described in the text.

To investigate the wheat floret ‘second opening’ process (Hoshikawa, 1960; Pickett, 1993), we utilized the genetic male-sterile mutant line *ms5* in the cultivar Chris background (Sasakuma *et al.*, 1978). Segregating male-fertile and male-sterile plants were used for physiological observations and measurements (Supplementary Fig. S1A). Plants were grown in a controlled growth room to minimize environmental effects on flower development. Time course observation of the flowering process was carried out (see details in the Materials and methods), starting from 1 DF (Zadoks scale 59) until 9 DF (Zadoks scale 69–70). In male-fertile plants, the glume and floret remain closed after anthesis and only a slight variation in the angle of the glume and floret was observed (Figs 1A, 2A; Supplementary S1B). Under these growth conditions, 3 DF was around the time of anthesis in male-fertile plants and anther extrusion was observed from the primary floret (Fig. 1A). The ‘first opening’ of florets at anthesis is induced by lodicule swelling, and this is normally short (up to 30 min) as mentioned above. In male-fertile plants, most florets we observed on 3 DF have collapsed lodicules (Fig. 1B), and florets were already closed again. Therefore, in male-fertile plants, the ‘first opening’ was not truly captured by glume and floret angle measurements, indicating that the angles showed little variation throughout the flowering period (Fig. 2A). In contrast, male-sterile plants started to open florets from 3 DF and were almost fully open by 5 DF (Figs 1A, 2A). Florets remained open until 9 DF and closed slowly after that.

During this flowering period, the grain develops vertically in the fertile floret by increasing in height and filling the space inside the floret (Fig. 1B, top panel). The stigma atrophied after pollination and the anthers may or may not be found in the floret after anthesis and extrusion. In male-sterile florets, ovary height showed a slight increase, but the ovary revealed a significant increase in radial size (Fig. 1B). The stigma remained feathery and intact by 7 DF, with atrophication initiating soon after under these growth conditions. Interestingly, the lodicule is already collapsed by 3 DF in the male-sterile florets with a minor but significant increase in glume and floret angles by 3 DF (Figs 1, 2A). Therefore, this does not represent the ‘first opening’ but the initial phase of the ‘second opening’, as it is not the effect of lodicule swelling but rather caused by another factor, most probably via the ovary.

### Ovary radial size increase in male-sterile plants opens up the wheat floret

A previous study reported that both lodicule swelling and an ovary size increase were observed upon the ‘second opening’ in male-sterile wheat florets (Hoshikawa, 1960). However, our observations in male-sterile plants revealed no observable influence of lodicules on the ‘second opening’, as mentioned above. In order to investigate the role of ovary size increase on the ‘second opening’, we dissected ovaries over the course of flowering and took three-dimensional measurements for height, width, and depth (Supplementary Fig. S1C). Because of the possibility that in male-fertile versus male-sterile plants, both the timing and rate of reproductive development may differ, we used the standard time from heading on the Zadoks scale (Zadoks *et al.*, 1974) as an indicator of flowering stage. Observations comparing male-fertile and male-sterile ovary size at 1 DF revealed no significant difference (Fig. 2B–E), suggesting that genetic male sterility has little effect on female reproductive development at this stage.

For height, the fertilized ovary showed a steady increase after pollination around 3 DF, and by 7 DF the difference between male-fertile and male-sterile plants became apparent (Fig. 2B). Both fertilized and unfertilized ovary width increased during the flowering period; however, the width of unfertilized ovaries from male-sterile plants was significantly larger than that of fertilized ovaries from male-fertile plants after 3 DF (Fig. 2C). An increase in ovary depth is rapid and more apparent in unfertilized ovaries of male-sterile plants, with ovary depth in male-sterile plants being significantly larger than that in male-fertile plants after 3 DF (Fig. 2D). A significant increase in both width and depth of the unfertilized ovary results in a radial expansion of the ovary into a conical shape (Fig. 2E). The increased ovary depth exceeds the breadth of lemma at its corresponding position (Supplementary Fig. S2). Unfertilized ovary depth kept increasing until 9 DF, coupled with only a slight increase in height. This showed the strongest correlation with floret angle (*r* = 0.92) among the three ovary size measurements. Therefore, ovary swelling in male-sterile plants induces the separation of the palea from the lemma, leading to floret opening.

### Emasculation also results in ovary swelling but is not a direct cause of induction

Male sterility in *ms5* is caused by unknown recessive genetic factors. A possibility is that the causal mutation may be responsible for induced ovary swelling and flower opening as male-sterile plants require open pollination for setting seed. We additionally investigated the independent wheat male-sterile mutant line *ms1* (Driscoll, 1975; Sasakuma *et al.*, 1978), and found ovary swelling and flower opening traits comparable with those observed for *ms5* (data not shown). Therefore, ovary swelling is unlikely to be associated with the specific genetic mutation for *ms1* or *ms5*, but is likely to be due to either an absence of fertile pollen or a lack of pollination. This was further confirmed by emasculation of male-fertile plants (Supplementary Fig. S3A). When anthers were removed at 1 DF in the spring wheat cultivar Excalibur, ovary height and depth at 7 DF were similar to those of unfertilized male-sterile *ms5* plants (Supplementary Fig. S3B). We confirmed ovary swelling by emasculation in >15 wheat cultivars (data not shown); therefore, this is not a cultivar-dependent trait.

**Fig. 3.**
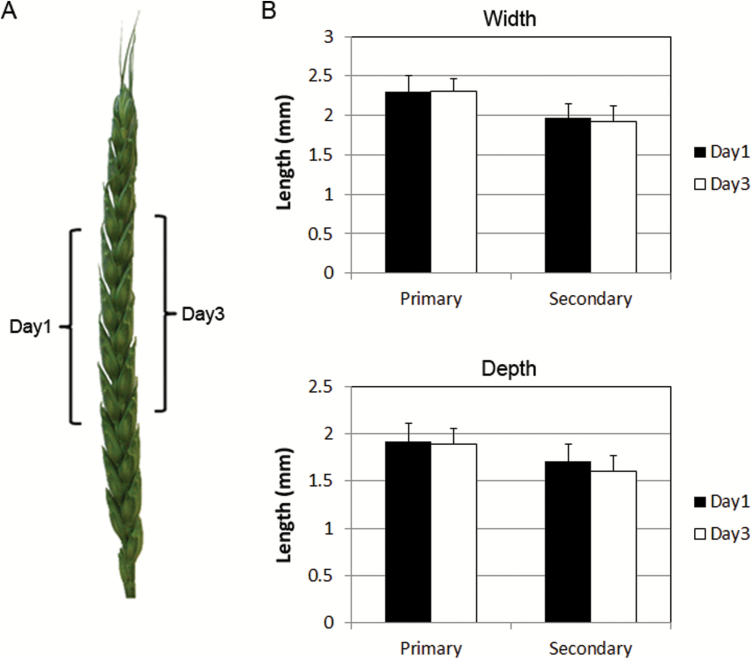
Effect of emasculation timing on ovary size. (A) Male-fertile *Ms5*/Chris spikelets were emasculated at two different time points. On one side of the spike, four middle spikelets were emasculated on 1 DF (Day1). Spikelets located on the opposite side of the same spike were emasculated early in the morning of 3 DF (Day3) before anthesis. (B) Ovary size measurement on 5 DF of samples emasculated at various flowering times. Ovary width and depth of primary and secondary florets are shown. No significant difference was found between samples emasculated on 1 DF and 3 DF. Error bars=SD.

Since the anther filament is connected to the base of the ovary (Supplementary Fig. S1D), it is conceivable that communication occurs between the anthers and ovary. The ovary may be able to detect the absence of fertile pollen due to male sterility or emasculation, leading to ovary swelling. To test this hypothesis, we emasculated anthers at 1 DF from the spikelets aligned on one side of the spike and at 3 DF from the spikelets on the opposite side of the same spike, and ovary size was measured at 5 DF (Fig. 3A). Under this hypothesis, early emasculation should lead to the induction of early ovary swelling. However, measurement of ovary size in primary and secondary florets did not show any significant difference in width and depth between ovary samples emasculated on 1 DF and 3 DF (Fig. 3B). Therefore, we concluded that the absence of fertile pollen or anther–ovary communication is unlikely to be a direct cue. It is reasonable to assume that the absence of pollination or fertilization may trigger the initiation of ovary swelling.

### Timing of anther filament elongation and initiation of ovary swelling

**Fig. 4.**
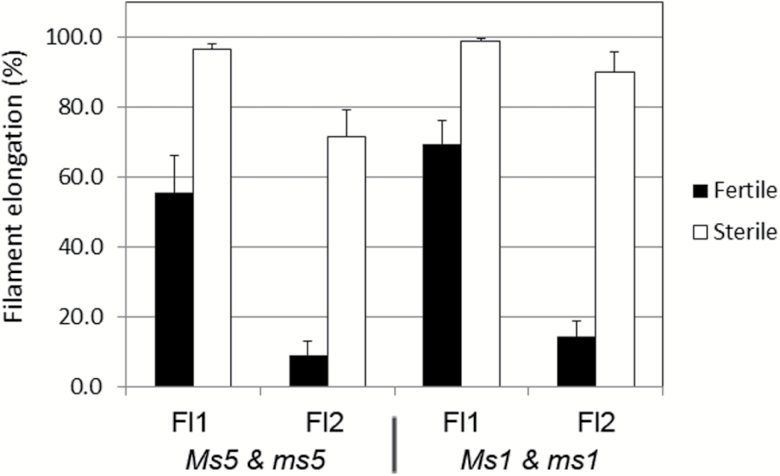
Timing of filament elongation in male-fertile and male-sterile plants. Primary (Fl1) and secondary (Fl2) florets of eight middle spikelets were opened during the late morning of 3 DF and were checked for filament elongation. The percentage of florets containing one or more elongated filaments in male-fertile and male-sterile plants is indicated on the *y*-axis. Two independent male-sterile mutant lines (*ms5* and *ms1*) were used for this experiment. There are significant differences (*t*-test, *P*-value <0.01) between fertile and sterile samples in all combinations. Error bars=SE.

We next addressed the question of how and when does the ovary detect the absence of pollination and begin to enlarge. Comparing the male-sterile *ms5* and male-fertile *Ms5* plants at 3 DF, we observed that the width and depth of male-sterile ovaries were already significantly larger than that of male-fertile ovaries (Fig. 2C, D). This indicated that in the male-sterile plants ovary swelling was already initiated by 3 DF. Furthermore, male-sterile *ms5* florets had also initiated opening by 3 DF (Figs 1A, 2A). More interestingly, we frequently noted that anther filaments were already elongated at 1 DF in male-sterile florets (Fig. 1B). Generally at anthesis in wheat, floret first opening driven by lodicule swelling, anther dehiscence, and anther filament elongation occur simultaneously, assisting self-pollination (Heslop-Harrison and Heslop-Harrison, 1996). Considering these pollination behaviours in wheat, one can make a reasonable assumption that it will be self-pollinated when anther filaments elongate. Therefore, we investigated the timing of filament elongation in relation to ovary size. We checked the frequency of filament elongation in the male-fertile *Ms5* and male-sterile *ms5* plants at 3 DF. In the primary floret (Fl1 in Fig. 4), about half of the florets had elongated filaments in male-fertile *Ms5* plants, while >95% of florets had elongated filaments in male-sterile *ms5* plants (Fig. 4). In the secondary floret (Fl2), <10% of male-fertile *Ms5* plants had elongated filaments, while in *ms5* plants ~70% of secondary florets had elongated filaments. Similarly, we found early filament elongation in the independent male-sterile line *ms1*.

Furthermore, we observed late filament elongation in the emasculated male-fertile samples, which normally occurred after 3 DF. In emasculated male-fertile *Ms5* samples, ovary depth at 5 DF for primary and secondary florets was <2 mm (Fig. 3B), while in male-sterile *ms5*, where the filament elongated as early as at 1 DF, the average ovary depth at 5 DF was >2.5 mm (Fig. 2D). Ovary width and depth at 5 DF in emasculated male-fertile *Ms5* samples was approximately the size of male-sterile *ms5* at 3 DF. This suggests that the ovary swelling process initiated later in emasculated male-fertile samples coincident with later filament elongation, compared with male-sterile *ms5* plants. Taken together, these results suggest that there is a positive correlation between timing of filament elongation and initiation of ovary swelling.

### Unfertilized ovary has intact and enlarged mesocarp cells

**Fig. 5.**
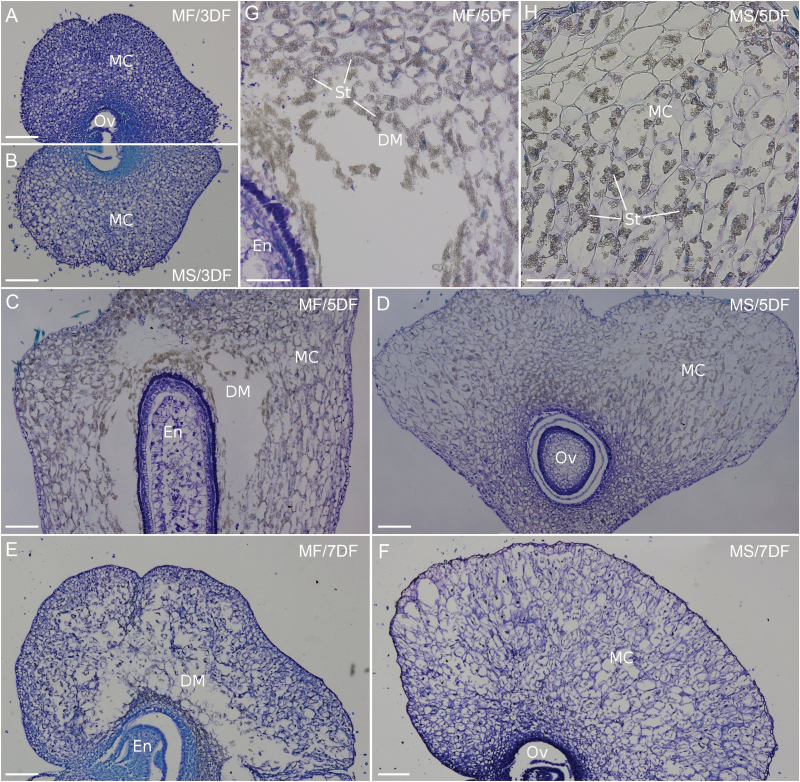
Histological examination of fertilized (MF; A, C, E, and G) and unfertilized (MS; in B, D, F, and H) ovary at different flowering stages. (A) and (B) Cross-sections of the top part of the ovary, proximal to the stigma, at 3 DF. (C) and (D) Longitudinal sections of the middle part of the ovary from the ventral (left) to dorsal (right) side at 5 DF. (E) and (F) Cross-sections of the top part of the ovary at 7 DF. (G) Magnified image of the area containing degenerating mesocarp cells in (C). (H) Magnified image of the dorsal part of the ovary in (D) containing enlarged mesocarp cells. Bars in (A) to (F)=200 µm and bars in (G) and (H)=100 µm. Abbreviations: DM, degenerating mesocarp cells; En, endosperm; MC, mesocarp cells; Ov, ovule; St, starch granules.

In order to understand the underlying mechanisms involved in ovary swelling, we carried out histological analyses of fertilized and unfertilized ovaries. The wheat ovary consists of the ovule, containing the embryo sac or developing embryo and endosperm, and pericarp which surrounds the ovule (Evers and Millar, 2002). The pericarp is composed of three parts: exocarp, mesocarp, and endocarp (Zhou *et al.*, 2009; Xiong *et al.*, 2013). At the early stages of caryopsis development, mesocarp is the major part of the pericarp, and the mesocarp contains >10 layers of parenchyma cells that are loosely arranged. At 3 DF, structural features of the mesocarp of male-fertile and male-sterile ovaries are similar (Fig. 5A, B). At 5 DF in the fertilized ovary, the developing endosperm was found to be located in the centre of the ovary and growing vertically. Mesocarp cells located in the top section of the ovary and close to the endocarp and endosperm showed typical degradation (Zhou *et al.*, 2009; Xiong *et al.*, 2013), as indicated by the presence of an empty space without any cell structures (Fig. 5C, G). In contrast, there was no seed development in the unfertilized ovary, and ovule size was unaltered, resulting in minor growth vertically (Fig. 5D). Mesocarp cells remained intact and were enlarged, especially in the top part of the ovary (Fig. 5H), including both ventral and dorsal sides (left and right, respectively, in Fig. 5D). At 7 DF, degradation of the mesocarp in the fertilized ovary was more extensive (Fig. 5E). This is consistent with the observation that all mesocarp cells eventually disappear during grain development while the exocarp remains as the outer layer of the seed coat for protection (Xiong *et al.*, 2013). Contrastingly, the mesocarp cells of the unfertilized ovary at 7 DF were still largely intact (Fig. 5F). Enlargement of ovary depth in the unfertilized ovary was not caused by an increase of mesocarp cell number but due to the increase of cell size (Supplementary Fig. S4). We also observed that a considerable number of starch granules were accumulated in the mesocarp cells in both fertilized and unfertilized ovaries (Fig. 5G, H).

**Fig. 6.**
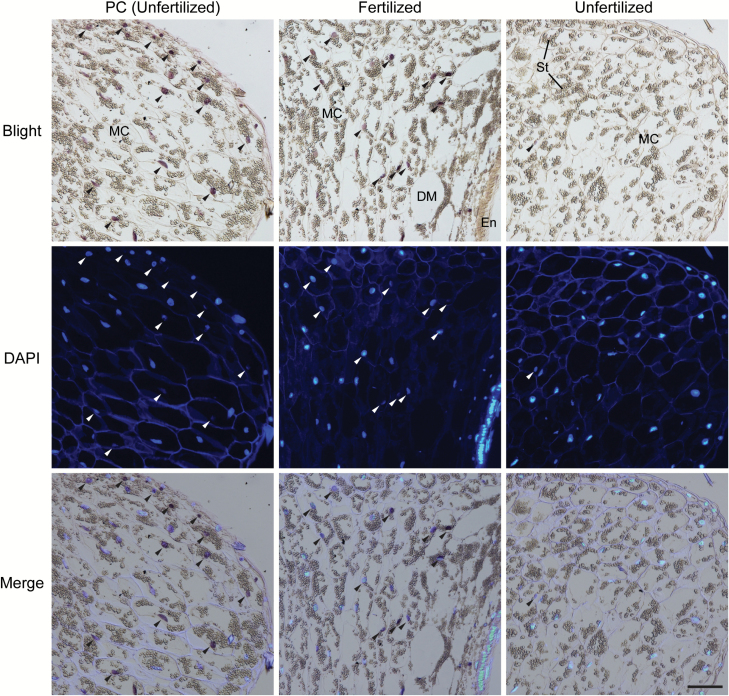
Detection of DNA fragmentation associated with programmed cell death in the ovary by TUNEL assay. Longitudinal sections of 5 DF ovary samples were used for the TUNEL assay. For positive control samples (PC), the section of unfertilized ovary was treated with DNase I prior to TUNEL assay (left column). Dark purple staining in the bright field image (top panels) indicates nuclei containing fragmented DNAs (arrowheads). DAPI-stained nuclei of the same sample slides are shown in the middle panel and merged images at the bottom. Nuclei stained dark in the TUNEL assay were difficult to stain with DAPI, and therefore showed a weaker DAPI signal. Scale bar=100 µm. Abbreviations: DM, degenerating mesocarp cells; En, endosperm; MC, mesocarp cells; St, starch granules.

Degradation of the pericarp in the fertilized ovary is the process of PCD that results in increased vacuolation, chromatin condensation, and nuclear DNA degradation (Zhou *et al.*, 2009). In barley and wheat, nuclear DNA fragmentation has been shown in the pericarp of developing grain by TUNEL assay (Zhou *et al.*, 2009; Radchuk *et al.*, 2011). For this reason, we performed TUNEL assays to investigate the fate of mesocarp cells in the unfertilized ovary. In this method, cells with fragmented nuclear DNA can be detected on the sections of ovary tissue by purple color staining. For example, in the positive control sample, unfertilized ovary sections pre-treated with DNase I showed dark stained nuclei in the pericarp (indicated by arrowheads in Fig. 6 left column). In 5 DF fertilized ovary, nuclei of mesocarp cells close to degenerating cells showed strong positive staining, indicating fragmentation of nuclear DNA (Fig. 6, middle column). In contrast, nuclei of mesocarp cells in unfertilized ovary at the same 5 DF stage rarely showed any staining, indicating a lack of PCD signatures (Fig. 6, right column). This is consistent with mesocarp cells in unfertilized ovary maintaining an intact status. In summary, the absence of seed development in an unfertilized ovary results in limited vertical growth of the ovary, while mesocarp cells remained intact and enlarge, leading to an increase in ovary radial size.

### Orthologues of *HvVPE4*, which is involved in barley pericarp PCD, are down-regulated in the unfertilized swelling ovary

Several plant proteases possess caspase-like activity and are shown to be frequently involved in plant PCD, including a vacuolar processing enzyme (VPE) (Hara-Nishimura *et al.*, 2005; Hatsugai *et al.*, 2006). In barley, seven *VPE* genes containing a caspase-1-like domain have been identified in developing grain (Radchuk *et al.*, 2011). Among those, *HvVPE4* is highly up-regulated in pericarp and specifically expressed in mesocarp cells, suggesting a role in the barley pericarp PCD. We identified wheat *HvVPE4* orthologues in chromosome group 5 by BLAST searches (Supplementary Fig. S5). Barley *HvVPE4* is located on chromosome 5HL, and this region is syntenic to wheat chromosome group 5. The barley and wheat *VPE4* sequences show high sequence identity at both the DNA (93–94%) and protein (94%) level. Phylogenetic analysis further confirmed that these wheat sequences are highly likely to be authentic barley *HvVPE4* orthologues (Supplementary Fig. S6). Therefore, we designated them *TaVPE4-A*, *TaVPE4-B*, and *TaVPE4-D*.

**Fig. 7.**
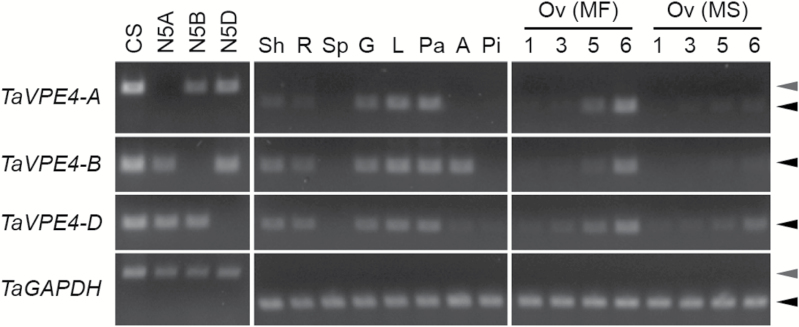
Expression of *TaVPE4* genes in the ovary and various other tissues. Gene expression was investigated by RT–PCR using homeologue-specific primers, which were developed and tested for specificity using nullisomic–tetrasomic line genomic DNAs (left panel). N5A, nullisomic 5A–tetrasomic 5D; N5B, nullisomic 5B–tetrasomic 5D; N5D, nullisomic 5D–tetrasomic 5B. Expression of *TaVPE4* genes in various tissues (middle panel). Tissues used were seedling shoot (Sh), seedling root (R), immature spike (Sp), glume (G), lemma (L), palea (Pa), anther (A), and pistil (Pi). Expression of *TaVPE4* genes in ovary tissues (right panel). Ovaries (Ov) from male-fertile (MF) and male-sterile (MS) plants were collected at 1, 3, 5, and 6 DF, and used for expression analysis. *TaGAPDH*, the wheat glyceraldehyde-3-phosphate dehydrogenase gene used for template amplification control. For *TaBPE4-A* and *TaGAPDH*, grey and black arrowheads indicate genomic DNA and cDNA fragment, respectively.

Gene-specific primers were designed for each homeologue and were validated for specificity by using wheat nullisomic–tetrasomic lines (Sears, 1966), in which one pair of the group 5 chromosomes is replaced by another group 5 chromosome pair (Fig. 7, left panel). We used semi-quantitative RT–PCR to investigate gene expression and found that all three homeologues were expressed in various tissues and showed similar patterns of tissue specificity, with minor variations (Fig. 7, middle panel). Expression in the ovary of male-fertile plants indicated that each *TaVPE4* homeologue showed low expression before anthesis (1 DF and 3 DF) but was up-regulated after pollination (5 DF and 6 DF) (Fig. 7, right panel). In contrast, in unfertilized ovaries of male-sterile plants, a low or basal level of *TaVPE4* expression was maintained at 5 DF and 6 DF stages. Our results show that the expression pattern of *TaVPE4* is consistent with features of mesocarp cell degradation and PCD in fertilized and unfertilized ovaries, and suggest that pollination and/or fertilization may induce *TaVPE4* activation.

### Emasculation of barley also causes ovary swelling

Among the species closely related to wheat, barley is also autogamous and a globally important cereal crop for food, feed, and malting (FAO, 2009). We investigated ovary swelling in the barley cultivar Bowman by emasculation. The shape of the barley ovary before anthesis is slightly different from that of wheat, being narrower and longer (Supplementary Fig. S7A). At 7 d post-emasculation, the top section of the barley ovary showed swelling and had the appearance of two fused spherical segments, whilst the bottom section remained relatively unchanged in shape (middle in Supplementary Fig. S7A). Vertical growth in the unfertilized/emasculated ovary was limited, compared with that of the developing grain (7 d post-anthesis). The observed similar ovary swelling patterns in wheat (Supplementary Fig. S2) and barley (Supplementary Fig. S7B) suggest a conserved mechanism for the ‘second opening’ in the absence of self-pollination.

## Discussion

### Flower opening in autogamous cereals

Flower opening in cereals at anthesis is caused by lodicule swelling and it has been well studied in autogamous cereals, such as rice, wheat, and barley (de Vries, 1971; Frankel and Galun, 1977; Virmani, 1994). The ‘first opening’ by lodicule swelling in wheat is most likely to be designated for anther extrusion, as it is short temporally but is sufficient time for the rapid anther filament elongation process (Heslop-Harrison and Heslop-Harrison, 1996). The barley *Cleistogamy 1* (*Cly1*) gene encodes an AP2 domain transcription factor and has a role in promoting lodicule development. Nucleotide substitution at the miR172 target site of *Cly1* results in small undeveloped lodicules, hence making it a cleistogamous floret (Nair *et al.*, 2010). There are three homeologous orthologues of *Cly1* in wheat, and they are predicted to have a similar function to barley *Cly1* (Ning *et al.*, 2013). The ‘first opening’ at anthesis induced by turgid lodicules is rather brief, and normally lasts <30 min in barley and wheat (de Vries, 1971; Kirby *et al.*, 1983; Pickett, 1993). To our knowledge, there is no information available relating to potential cross-pollination rates that could occur during the ‘first opening’ period, but the opportunity is short. We demonstrated here that the unfertilized ovary has a role in re-opening the wheat flower after anthesis, an event known as the ‘second opening’. Swollen ovaries and the resultant floret opening are maintained for several days until the stigmas atrophy and lose receptivity (Figs 1, 2; Supplementary Fig. S1E). This provides a longer opportunity in wheat for outcrossing when initial self-pollination fails. The ‘second opening’ induced by ovary swelling is also found in the autogamous cereal barley (Supplementary Fig. S6; Kirby *et al.*, 1983), suggesting a conserved mechanism in these species.

Rice is another autogamous cereal that has been studied extensively for flowering biology and pollination behaviour (Namai and Kato, 1988; Taillebois and Guimaraes, 1988; Virmani, 1994). Rice also exhibits a first opening event with brief flower opening and closure (50–90 min period) associated with lodicule swelling and withering (Parmar *et al.*, 1979; Virmani, 1994). However, prolonged opening of rice flowers associated with genetic factors has been reported in a few rice genotypes (Kondo and Ishiki, 1933; Nagao and Hoshika, 1938; Parmar *et al.*, 1979), including the *OsJAR1* mutant (Riemann *et al.*, 2008; Xiao *et al.*, 2014). *OsJAR1* encodes jasmonic acid (JA) synthase, involved in a JA signalling pathway, and a loss of function of *OsJAR1* delayed lodicule withering, leading to florets remaining open for several days. Prolonged flower opening is also known in cytoplasmic male-sterile (CMS) rice plants (Parmar *et al.*, 1979; Parmar *et al.*, 1980; Taillebois and Guimaraes, 1988). In CMS rice Wu10A, development of pseudograins, which are enlarged ovaries without fertilization, has been reported. Pseudograins accumulate soluble sugars and free amino acids, and consequently the enlarged filling causes the rice husk to crack (Chaudhary, 1982). It needs to be elucidated whether the prolonged flower opening period in CMS rice is associated with prolonged lodicule swelling or the change in ovary shape and size. It would be interesting to see if the ‘second opening’ mechanism is conserved in the three autogamous cereal species wheat, barley, and rice.

### A model for the ‘second opening’ in wheat

**Fig. 8.**
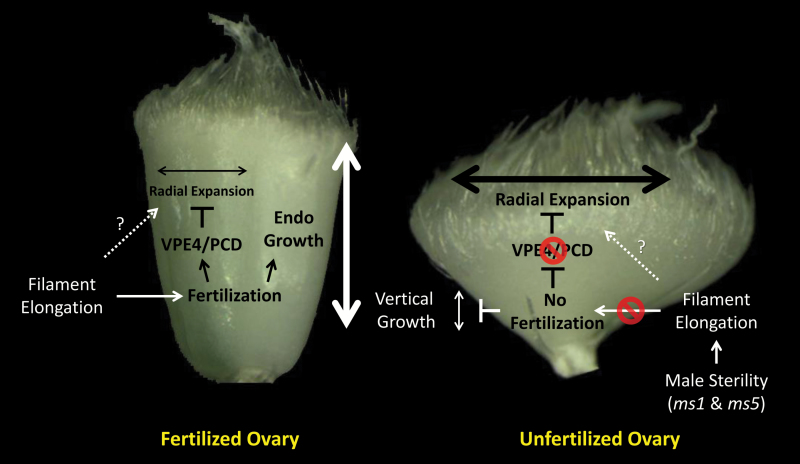
A model for the wheat ‘second opening’ process by ovary swelling.

Figure 8 summarizes our findings of the ‘second opening’ process in wheat florets. Soon after filament elongation, one would expect the ovary to undergo self-pollination. However, this may not occur when anthers do not contain viable pollen, and hence the ovary may opt for an alternative mode of pollination. Our results suggest that filament elongation is temporally associated with the initiation of ovary swelling. This would allow wheat florets to undergo a seamless transition in pollination strategies from selfing to open pollination without compromising stigma receptivity. An ovary swelling or radial size (width and depth) increase also occurred in the fertilized ovary (Fig. 2C, D). However, this is somewhat offset by degradation of mesocarp cells via PCD, possibly associated with up-regulation of *TaVPE4* expression (Fig. 8, left panel), resulting in limited radial size growth. In addition, the maximum ovary depth position moves up as grains develop vertically, therefore the developing wheat grain, forming an ellipsoid shape, is encased between the palea and lemma which has a wider breadth at the middle position. In the unfertilized ovary, there is only a slight height increase due to the absence of fertilization. Mesocarp cells become enlarged and the pericarp increases in width and depth to the extent of exceeding the breadth of the lemma (Fig. 8, right panel). The enlargement is associated with an absence of PCD and mesocarp degradation, accompanied by a low or basal level of *TaVPE4* expression. Mesocarp cells in the pericarp rapidly synthesize and accumulate starch soon after fertilization, and later nutrients and sugars digested from the starch are translocated to the developing endosperm (Zhou *et al.*, 2009; Xiong *et al.*, 2013). We observed a considerable accumulation of starch in mesocarp cells of unfertilized ovaries like that found in the fertilized ovary (Fig. 5G, H). Absence of endosperm development and PCD in the mesocarp of the unfertilized ovary is likely to prevent or reduce starch translocation from the mesocarp, and this could partly contribute to the observed enlargement of these cells. In summary, facilitation of flower opening is another role for the pericarp, in addition to ovule protection and nutrient transportation and temporary storage (Chevalier and Lingle, 1983).

### Importance of cross-pollination in wheat

Improving cross-pollination efficiency is a critical factor in reducing the cost of hybrid seed production. Current and previous key research focus for wheat hybrid breeding has been directed to pollinator traits and pollination biology (de Vries, 1971, 1972, 1973, 1974*a*, *b*; Pickett, 1993; Langer *et al.*, 2014). Floret opening is a trait that has been studied in relation to spikelet position within the spike, spikelet density, and glume stiffness, but not in conjunction with the ovary size. Recently, Guo *et al.* (2015) demonstrated that variation in wheat ovary size is a heritable trait that is under strong genetic control. Thus, a relationship between the ovary/floret size ratio and flower opening angle in various wheat genotypes would be interesting to explore further in the context of hybrid breeding. Furthermore, nicking (i.e. optimal flowering coincidence) is one of the most critical factors for successful hybrid seed production (Porter *et al.*, 1965; Johnson *et al.*, 1967; Khan *et al.*, 1973). Our observations suggested that male-sterile plants might behave differently in reproductive development and timing of anthesis compared with the male-fertile segregants. It may require additional detailed observations of flowering behaviour of independent male-sterile lines for precision matching of anthesis of the male parent and floret opening of the male-sterile female parent.

Since wheat is a strong autogamous plant, failure of self-pollination causes significant yield loss, and male sterility is one of the major causes of this loss. The anther and microspores are more sensitive to environmental stimuli than female reproductive organs, especially around the time of meiosis, leading to partial or complete male sterility. On the other hand, the ovary is more resilient to some stresses such as drought and cold; therefore, it recovers from damage and set seed if pollinated with viable pollen (Saini and Aspinall, 1981; Paulsen and Heyne, 1984; Sohroyer *et al.*, 1995; Saini and Westgate, 1999; Ji *et al.*, 2010). Those ovaries that have missed self-pollination could be seen as a potential loss of resources. This resource could provide an opportunity to turn potential loss into gain by facilitating cross-pollination in inbred modern wheat cultivars. In conclusion, open florets in wheat with an unpollinated ovary represent a survival mechanism for autogamous cereals to set seed in the absence of self-fertilization and increase genetic diversity by cross-pollination.

## Supplementary data

Supplementary data are available at *JXB* online.

Fig. S1. Spike images and floral traits measured in this study.

Fig. S2. Measurement of the breadth of the lemma where the ovary is located.

Fig. S3. Ovary swelling caused by emasculation.

Fig. S4. Cell number and size for ovary pericarp cells.

Fig. S5. Alignment of amino acid sequences of barley HvVPE4 and orthologous wheat TaVPE4 homeologues.

Fig. S6. Phylogenetic tree of wheat TaVPE4s and other plant VPEs.

Fig. S7. Ovary swelling in barley caused by emasculation.

Table S1. Primers used for gene expression analysis by reverse transcription–PCR.

supplementary Figures S1-S7 and Table S1
