# Supplementary material for: Unfertilized ovary pushes wheat flower open for cross-pollination
Source: J Exp Bot. 2017 Nov 30;69(3):399–412. doi: 10.1093/jxb/erx410 (PMC5853862; doi:10.1093/jxb/erx410)
Supplement: supplementary Figures S1-S7 and Table S1 [file erx410_suppl_supplementary-figures-table.pdf]

**Supporting Information**

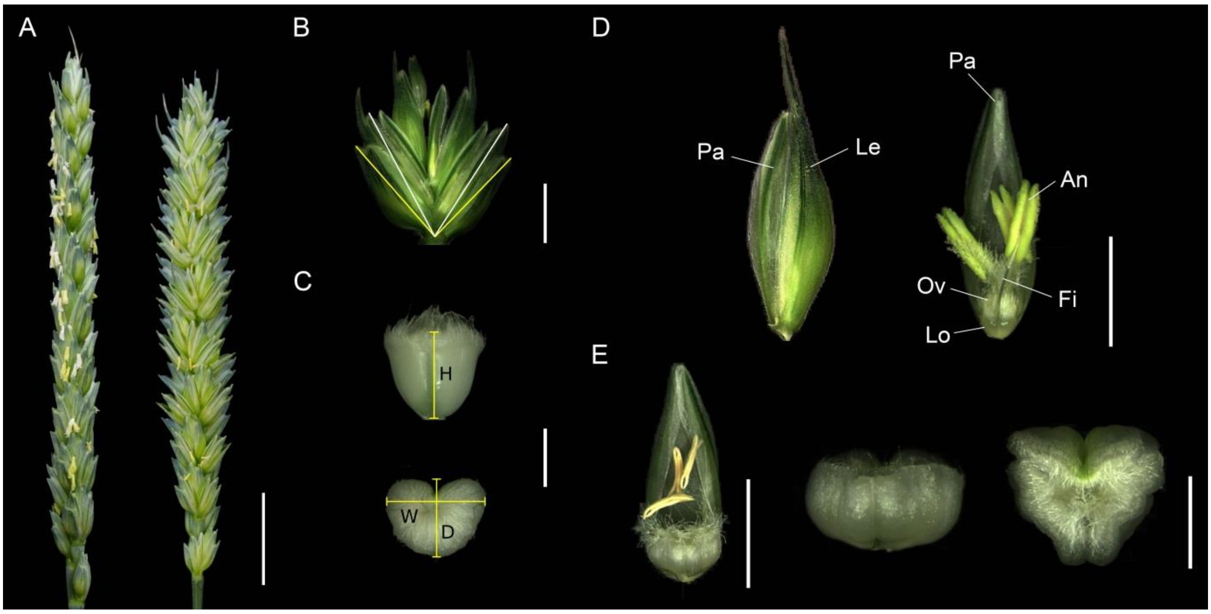


**Figure S1**. Spike images and floral traits measured in this study. (**A**) Male fertile (*Ms5*, left) and sterile (*ms5*, right) spike. (**B**) Glume and floret angles are indicated by yellow and white lines respectively. (**C**) Measurement of ovary size for height (H), width (W) and depth (D) relative to the axis displayed. Ovary height was measured from frontal images (top) which were taken by placing the palea (ventral) side of the ovary down and lemma (dorsal) side facing up towards the camera. Ovary width and depth were measured (bottom) by placing the rachis proximal side of the ovary down and stigma side facing up towards the camera. (**D**) Image of floret and reproductive organs in the floret. (**E**) Floret and ovary images of male sterile plant *ms5* at 11 DF. Abbreviations: An, anther; Fi, filament; Le, lemma; Lo, lodicule; Pa, palea; Ov, ovary. Bars in (**A**) = 2 cm, (**B**) = 5 mm, (**C**) = 2 mm, (**D**) = 5 mm, (**E**) = 5 mm and 2 mm for left and right, respectively.

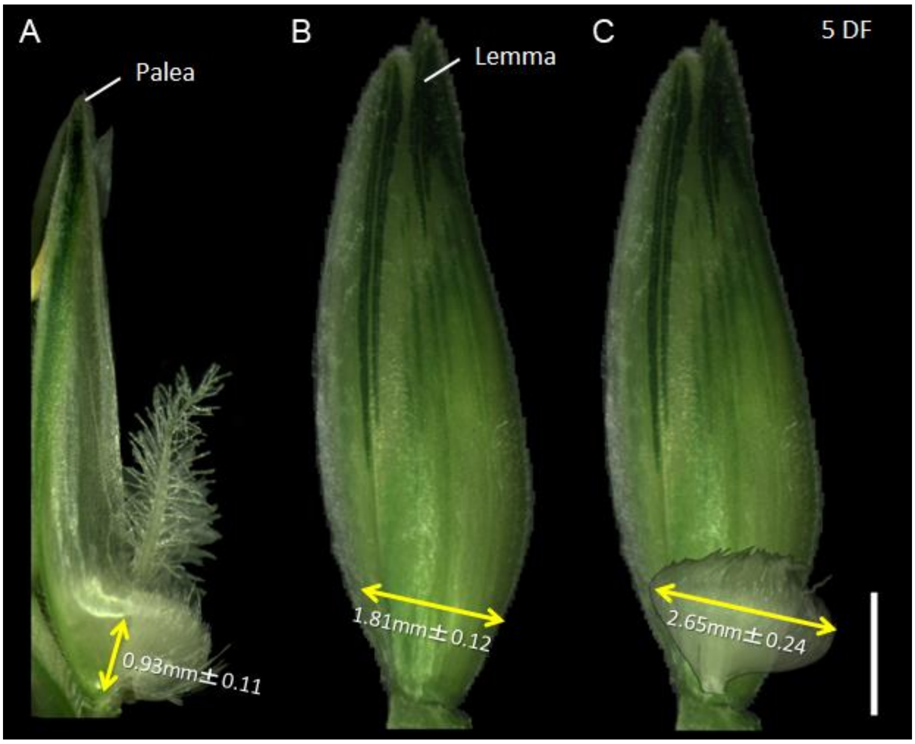


**Figure S2**. Measurement of breadth of lemma where the ovary is located. Breadth of lemma for primary florets (n = 10) of male fertile *Ms5* plants at 5 DF was measured from a side image. (**A**) Side image of male sterile *ms5* floret with lemma removed. Position of the maximum ovary depth was 0.93±0.11 mm proximal to the rachis. (**B**) Breadth of the lemma of male fertile *Ms5* at the corresponding position was 1.81±0.12 mm (**C**) A superimposed image of the swollen ovary of male sterile *ms5* (average ovary depth at 5 DF was 2.65±0.24 mm in **Fig. 2D**) over the lemma of male fertile *Ms5*. Bar = 2 mm.

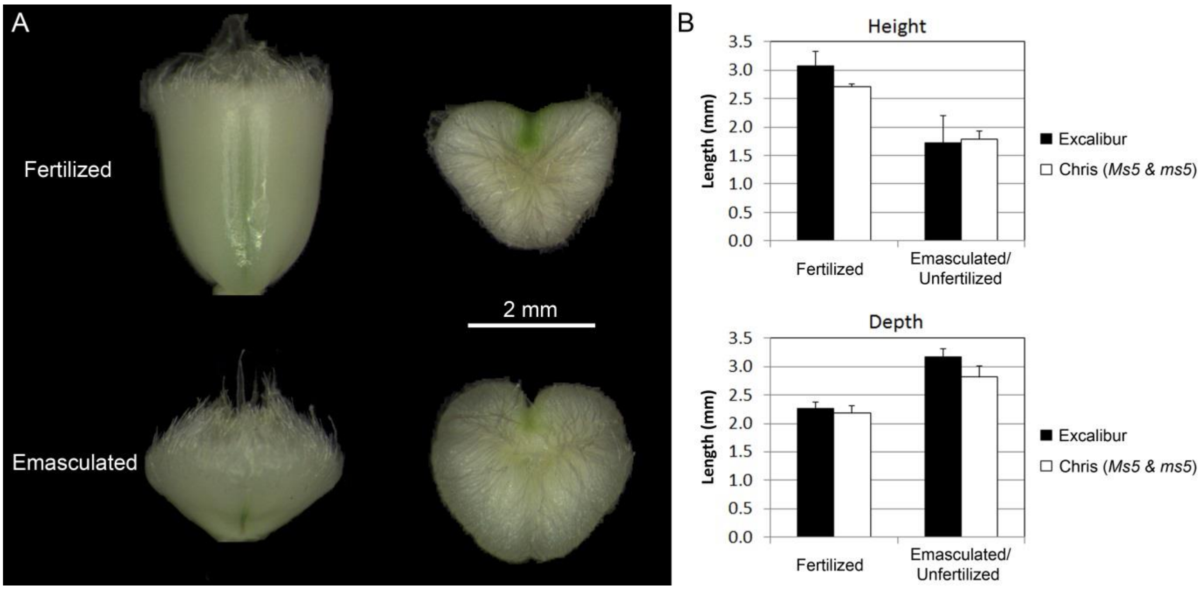


**Figure S3**. Ovary swelling caused by emasculation. (**A**) Front and top image of fertilized and emasculated ovaries of the wheat cultivar Excalibur. For emasculation, anthers were removed at 1 DF and ovary images were taken for measurements at 7 DF. (**B**) Height and depth of ovary of fertilized and emasculated samples from Excalibur were compared with 7 DF data previously obtained for male fertile *Ms5* and sterile *ms5* (in **Fig. 2**). No significant difference was found between fertilized Excalibur and fertilized *Ms5* or between emasculated Excalibur and unfertilized *ms5* ovary samples. Error bars, s.d.

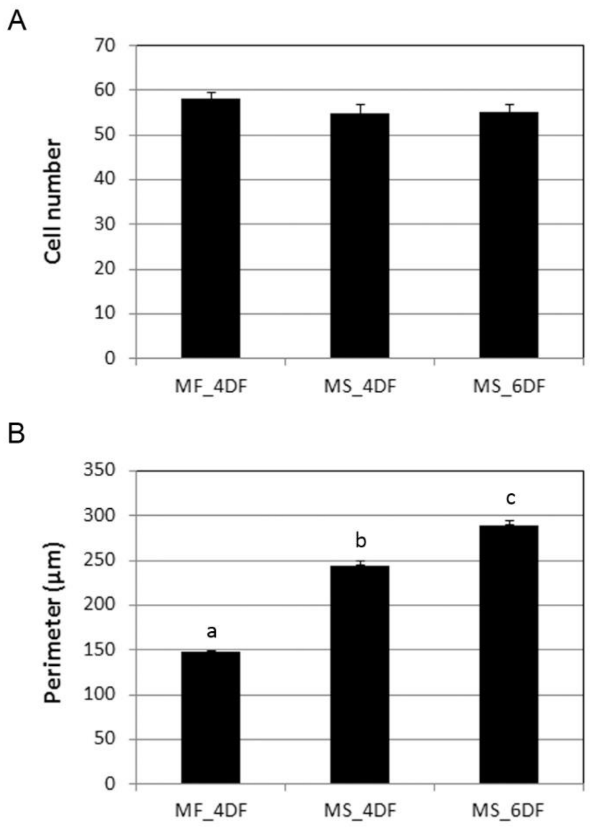


**Figure S4**. Cell number and size for ovary pericarp cells. (**A**) Number of pericarp cells aligned on the ovary depth orientation was counted in the longitudinal sections of fertilized ovary at 4 DF (MF_4DF), unfertilized ovary at 4 DF and 6 DF (MS_4DF and MS_6DF, respectively). No significant difference was detected between samples. (**B**) Measurement of perimeter of pericarp cells located at top dorsal position in the longitudinal section, as shown in **Fig. 5H**. Significant difference was found between samples (*t*-test, *p*-value <0.001). Error bars, s.e.m.

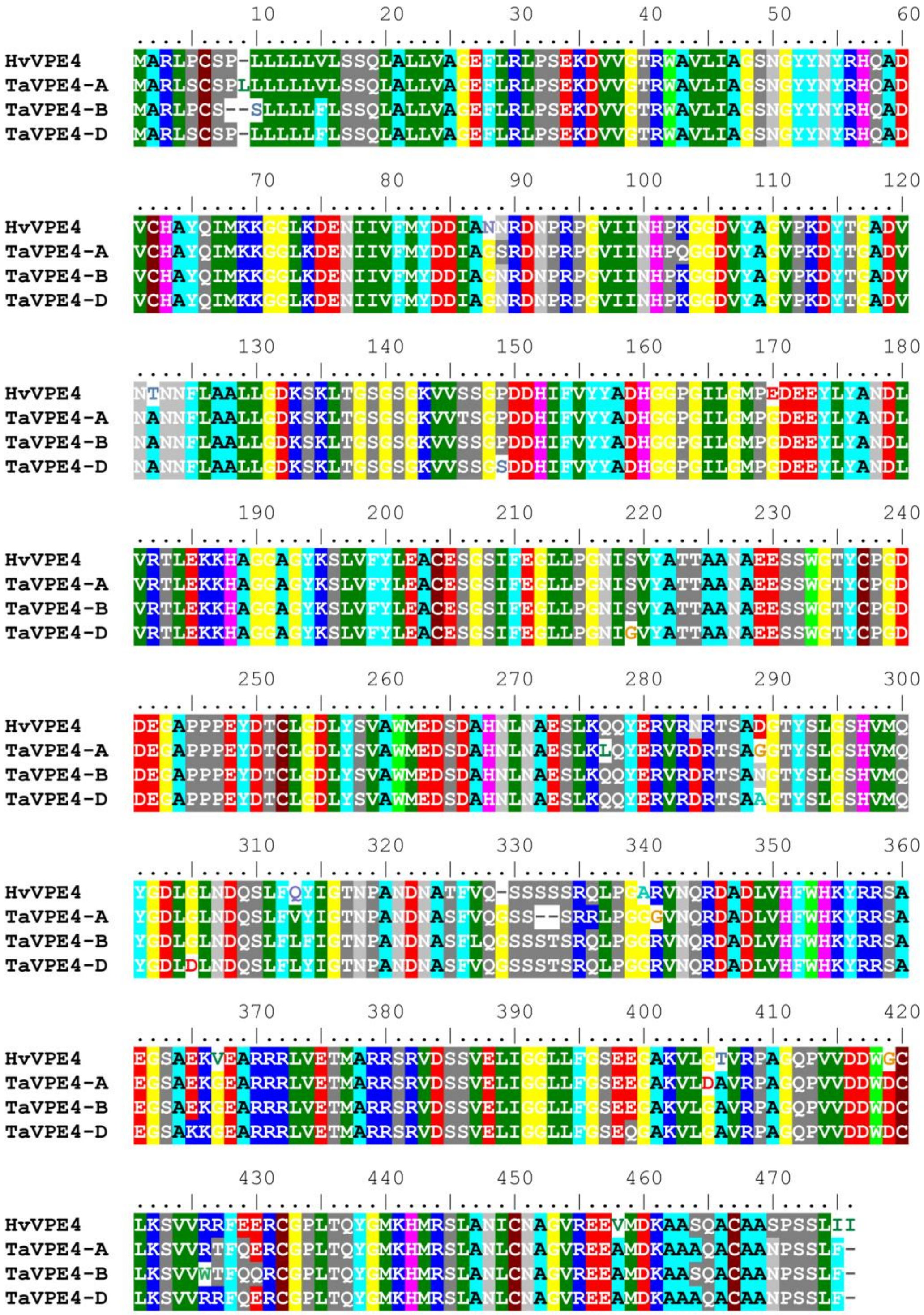


**Figure S5**. Alignment of amino acid sequences of barley HvVPE4 and orthologous wheat TaVPE4 homeologues. Deduced amino acid sequences of wheat TaVPE4 homeologues were obtained by translation of genomic sequences and high confidence predicted gene sequences.

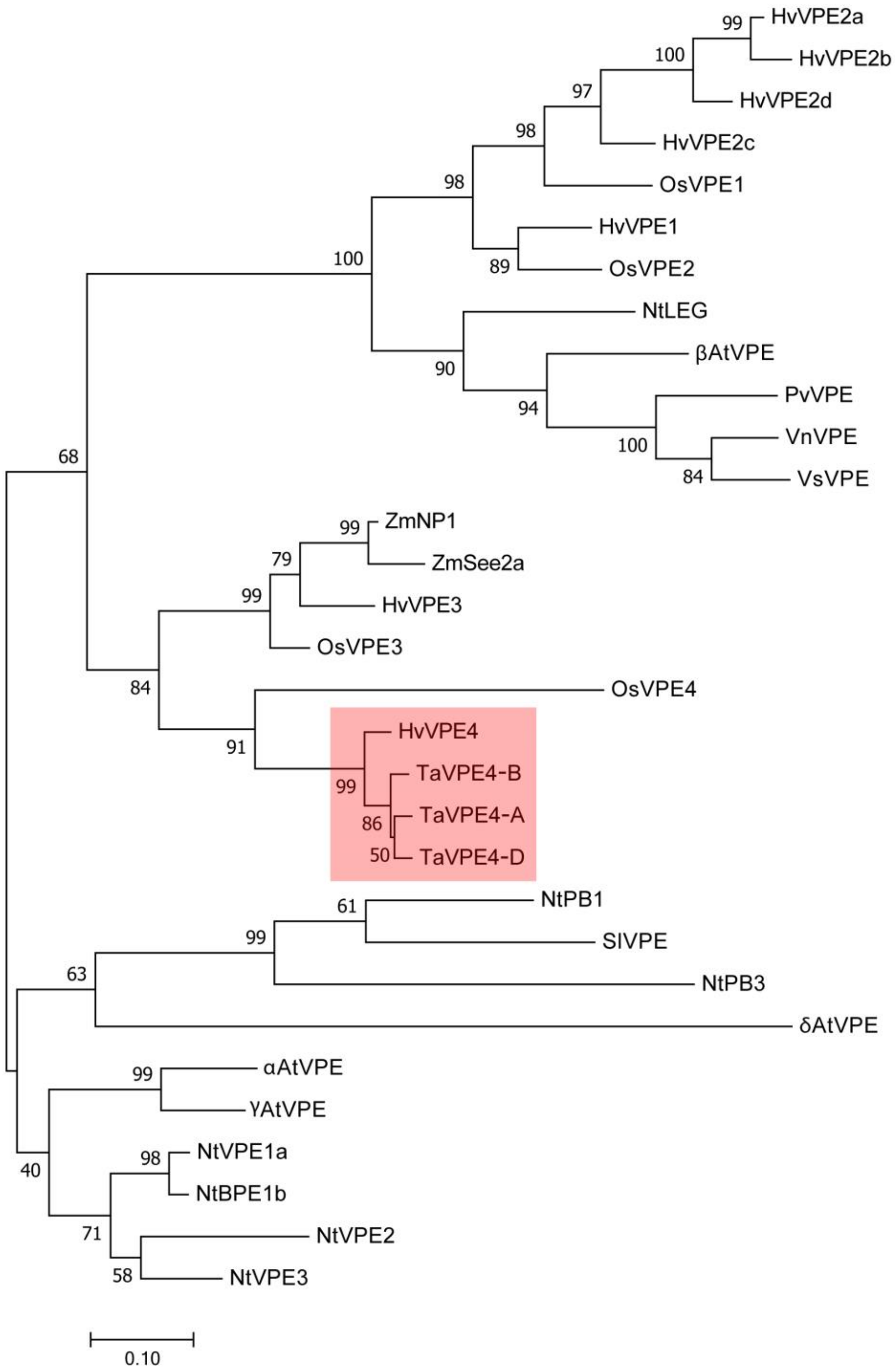


**Figure S6**. Phylogenetic tree of wheat TaVPE4s and other plant VPEs. The evolutionary history was inferred by using the Maximum Likelihood method based on the JTT matrix-based model (*Jones et al.*, 1992). The tree with the highest log likelihood (-6743.70) is shown. The percentage of trees in which the associated taxa clustered together is shown next to the branches. The tree is drawn to scale, with branch lengths measured in the number of substitutions per site. The analysis involved 31 VPE amino acid sequences. GenBank accession numbers of the sequences are as follows: *Arabidopsis thaliana*, αAtVPE (At2g25940), βAtVPE (At1g62710), γAtVPE (At4g32940), δAtVPE (At3g20210); *Nicotiana tabacum*, NtLEG (CAB42651),

NtPB1 (CAB42650), NtPB3 (CAE84598), NtVPE1a (BAC54827), NtVPE1b (BAC54828), NtVPE2 (BAC54829), NtVPE3 (BAC54830); Oryza sativa, OsVPE1 (Os04g0537900), OsVPE2 (Os02g0644000), OsVPE3 (Os01g0559600), OsVPE4 (Os05g0593900), OsVPE5 (Os05g0593900); *Phaseolus vulgaris*, PvVPE (O24326); *Solanum lycopersicum* SlVPE (CAB51545); *Vicia narbonensis* VnVPE (CAB16318); *Vicia sativa*, VsVPE (CAA07639); *Zea mays*, ZmNP1 (AAD04883), ZmSee2a (CAB64544). All positions containing gaps and missing data were eliminated. Evolutionary analyses were conducted in MEGA7 (Kumar *et al.*, 2016). The clade of HvVPE4 and TaVPE4s was highlighted in pink shade.

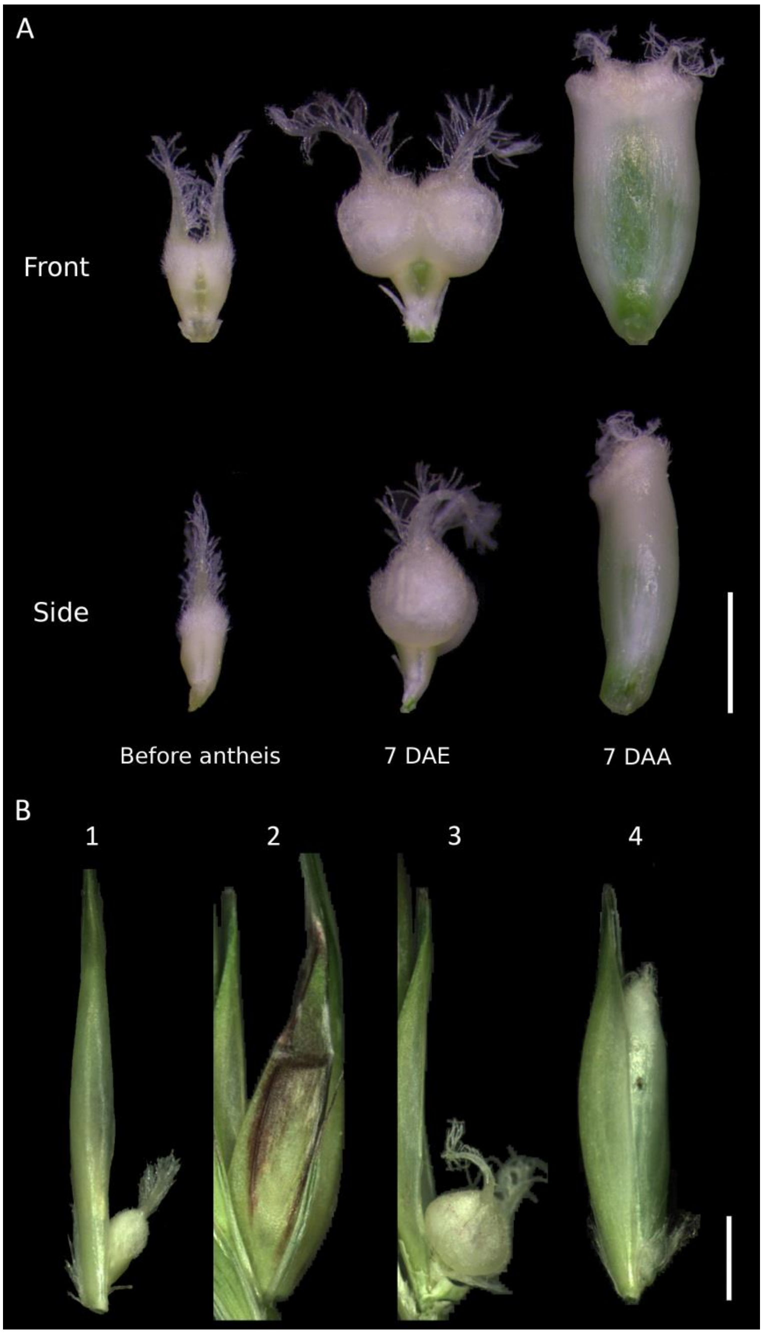


**Figure S7**. Ovary swelling in barley caused by emasculation. (**A**) Barley cv. Bowman ovary prior to anthesis at the time of emasculation (left). Unfertilized ovary 7 days after emasculation (7 DAE, middle). Fertilized ovary 7 days after anthesis (7 DAA, right). (**B**) Ovary swelling and floret opening in barley. Dissected floret before anthesis, lemma removed (**1**), opened floret induced by ovary swelling (**2**), swollen ovary in the floret, lemma removed (**3**) and fertilized and developing seed in barley floret (**4**). Bars = 2 mm.

**Table S1.** Primers used for gene expression analysis by reverse transcriptase-PCR.

| Table S1. Primers used for gene expression analysis by reverse transcriptase PCR. | | | | | |
|---|---|---|---|---|---|
| Gene Name | Homeologue | Forward | Reverse | Genomic fragment (bp) | cDNA fragment (bp) |
| *TaVPE-5A* | 5AL | CAGCGGCAAGGTCGTCAC | AGGCCTCCAGGTAGAAGACG | 299 | 191 |
| *TaVPE4-5B* | 5BL | CGGACGTCGGCCAACG | GTCCCGCTGGTTCACCCT | 188 | 188 |
| *TaVPE4-5D* | 5DL | CTCCTCTTCGGCTCTGAGC | CAGAGGTTGGCGAGCGAC | 173 | 173 |
| *TaGAPDH* | 6A, 6B, 6D | AGGGTGGTGCCAAGAAGGTCA | TATCCCCACTCGTTGTCGTA | 1143-1146 | 621 |
